# A Strategy to Reduce Critical Cardiorespiratory Alarms due to Intermittent Enteral Feeding of Preterm Neonates in Intensive Care

**DOI:** 10.2196/ijmr.7756

**Published:** 2017-10-20

**Authors:** Rohan Joshi, Carola van Pul, Anouk Sanders, Hans Weda, Jan Willem Bikker, Loe Feijs, Peter Andriessen

**Affiliations:** ^1^ Department of Industrial Design Eindhoven University of Technology Eindhoven Netherlands; ^2^ Department of Clinical Physics Máxima Medical Center Veldhoven Netherlands; ^3^ Department of Applied Physics Eindhoven University of Technology Eindhoven Netherlands; ^4^ Department of Neonatology Máxima Medical Center Veldhoven Netherlands; ^5^ Department of Patient Care & Measurements Philips Research Eindhoven Eindhoven Netherlands; ^6^ Consultants in Quantitative Methods CQM BV Eindhoven Netherlands; ^7^ Department of Pediatrics, Maastricht University Medical Center Faculty of Health,Medicine and Life Sciences School for Mental Health and Neuroscience Maastricht Netherlands

**Keywords:** preterm infants, enteral feeding, bradycardia, hypoxia, alarms

## Abstract

**Background:**

Many preterm infants require enteral feeding as they cannot coordinate sucking, swallowing, and breathing. In enteral feeding, milk feeds are delivered through a small feeding tube passed via the nose or mouth into the stomach. Intermittent milk feeds may either be administered using a syringe to gently push milk into the infant’s stomach (push feed) or milk can be poured into a syringe attached to the tube and allowed to drip in by gravity (gravity feed). This practice of enteral feeding is common in neonatal intensive care units. There is, however, no evidence in the literature to recommend the use of one method of feeding over the other.

**Objective:**

The aim of this study was to investigate which of the two methods of feeding is physiologically better tolerated by infants, as measured by the incidence of critical cardiorespiratory alarms during and immediately after feeding.

**Methods:**

We conducted a prospectively designed observational study with records of all feeding episodes in infants of gestational age less than 30 weeks at birth and with a minimum enteral intake of 100 mL/kg/day. In total, 2140 enteral feeding episodes were noted from 25 infants over 308 infant-days with records for several characteristics of the infants (eg, gestational age), feeding (eg, the position of infants), and of nursing-care events before feeding (eg, diapering). Logistic regression with mixed effects was used to model cardiorespiratory alarms for the push and gravity methods of feeding.

**Results:**

After adjustments were made for all confounding variables, the position of infants was found to be statistically significant in changing the outcome of critical alarms for the two methods of feeding (*P*=.02). For infants in the lateral position, push feeds led to 40% more instances of one or more critical cardiorespiratory alarms in comparison with the gravity method. Both methods of feeding created a statistically comparable number of alarms for infants in the prone position.

**Conclusions:**

This study provides objective data that may assist in optimizing enteral feeding protocols for premature infants. The incidence of critical cardiorespiratory alarms for infants in the lateral position can be lowered by the use of gravity instead of push feeding. No differences were observed between the two types of feeding when infants were in the prone position.

## Introduction

Every year, approximately 15 million infants are born prematurely (before 37 weeks of gestation), and this number is increasing [1]. Many of these infants require extensive medical attention and need to be admitted to neonatal intensive care units (NICUs). As these infants may be unable to coordinate sucking, swallowing, and breathing, milk feeds (expressed breast milk or formula) are delivered into the stomach via a tube passed through the nose or the mouth. This is known as enteral feeding and is common across NICUs [2].

Typically, enteral feeding can be *continuous feeding* delivered through the course of the day via an infusion pump or *intermittent feeding* usually administered over 10 to 20 min multiple times daily, for example, at intervals of 2 or 3 hours [2]. A Cochrane review found no differences between these two methods of enteral feeding for infants to achieve full feeds, days to discharge, or the incidence of necrotizing enterocolitis in preterm infants weighing less than 1500 g [2]. Although the theoretical risks and benefits for both methods have been proposed, clinical evidence supporting one method over the other is limited, and the choice of feeding is likely dependent on unit preferences and tradition [2]. Notably, though in a study of 33 preterm infants, fed once by each method, intermittent feeding was associated with lower apnea and apnea-related hypoxia [3].

Intermittent feeding is typically of two types—the *push* type is where the liquid is gently pushed through a syringe into the infant’s stomach, and the *gravity* type is where the liquid in the syringe is allowed to drip in under the influence of gravity. In the push method of feeding, small volumes of milk might be pushed at multiple times for each feed, whereas in gravity, the height of the syringe determines the rate of milk flow. A Cochrane review comparing push and gravity methods of feeding in preterm and low-birth-weight infants could not find evidence to recommend one method over the other [4]. Therefore, the method of delivering feeds is largely dependent on the preference of individual nurses, parents, and unit traditions.

In a recent study of nearly 600 preterm infants, an analysis of approximately 300,000 critical desaturations (oxygen saturation, SpO_2_ ≤80%) and 100,000 critical bradycardia (heart rate ≤80 beats per minute [bpm]) alarms showed a remarkable 2-hourly periodic increase in alarms [5]. This periodicity coincided with the timing and frequency of the enteral feeding routine in that unit. Some of the enteral feeding episodes were, as a matter of routine protocol, preceded by nursing care events, such as diapering, weighing, or nasopharyngeal suctioning, leading the authors to hypothesize that enteral feeding leads to cardiorespiratory instability resulting in critical patient monitor alarms around feeding [5]. However, the influence of nursing care itself cannot be definitively ruled out, as nursing care is also known to be a stressor [6-10].

This study is motivated by the hypothesis that enteral feeding leads to cardiorespiratory instability, by the prevalent clinical impression that cardiorespiratory events increase after feeding, and by the research recommendations of a Cochrane review to identify evidence-based strategies for the enteral feeding of preterm infants [4,5,11]. We used a data-driven approach to explore the relationship between feeding and cardiorespiratory alarms and to investigate which of the two methods of enteral feeding, push or gravity, is better tolerated by infants as measured by the prevalence of critical desaturation (SpO_2_ ≤80%) and bradycardia (heart rate ≤80 bpm) alarms measured during and immediately after feeding. This analysis accounts for multiple confounding variables such as the duration of feeding, the quantity of milk, the position of the infant, and any nursing care events that might have occurred immediately before feeding.

## Methods

### Patient Population

This prospectively designed observational study was conducted in the NICU of the Máxima Medical Centre, the Netherlands (level III; tertiary care NICU; single-room design) between October 2015 and June 2016. Preterm infants born at less than 30 weeks of gestation were eligible for the study once they were receiving an enteral feeding intake of at least 100 mL/kg/day. Several characteristics of the study group are presented in Table 1. According to routine clinical protocol, all infants were fed at 2-hourly intervals (at even hours) via an orogastric (in the case of binasal continuous positive airway pressure) or a nasogastric tube, both of which could stay in place for up to 4 weeks. It should be noted that the enteral feeding tube was not repeatedly inserted and removed but instead stayed in place until it required replacement. During the study period (308 infant-days), infants received routine need-based medication (eg, all infants receive caffeine), respiratory support, and therapeutic repositioning. As this study was performed using deidentified data corresponding to routine patient monitoring, a waiver (registered as N 2016.125) was provided by the local ethical committee in accordance with the Dutch law on medical research with humans (wet medisch-weten- schappelijk onderzoek,WMO).

**Table 1:** Characteristics of the patient population.

Characteristics	Median (IQR^a^)
Gestational age, weeks	27.5 (26.2-29.0)
Birth weight, g	965 (772-1116)
PMA^b^ during feeding episodes, week	31.3 (29.8-32.6)
PMA at discharge, week	33.6 (32.1-36.2)
Length of stay, day	40 (22-70)
Number of feeding episodes	77 (55-114)

^a^IQR: interquartile range.

^b^PMA: postmenstrual age, which is gestational age plus postnatal age.

### Enteral Feeding

For each feeding episode, nurses recorded the type of feeding (push or gravity), the type of milk (expressed breast milk or formula), the quantity of milk, the start and end time of feeding based on monitor time, the type of nursing care administered immediately before feeding, and the position of the infant (prone, lateral, or supine; 90% of lateral positions were additionally annotated as left or right lateral, whereas the remainder were merely annotated as lateral). According to the protocol, all nursing care events were performed before feeding, including changing the position of the infant. The types of nursing care records included diaper change, washing, weighing, airway suctioning, change of nasal mask or nasal prong, enteral administration of medication, miscellaneous care, and no care. After nursing care, enteral feeding was initiated. Gravity feeding was performed through a feeding tube (Vygon polyurethane Ch 6) connected to an open 10- to 20-mL syringe, placed approximately 15 cm above the patient to ensure forward flow. Push feeding was performed through the feeding tube connected to a closed 10- to 20-mL syringe by regularly pushing the milk through at a velocity of 1 to 2 mL/min.

### Patient Monitor Alarms

**Figure 1:**
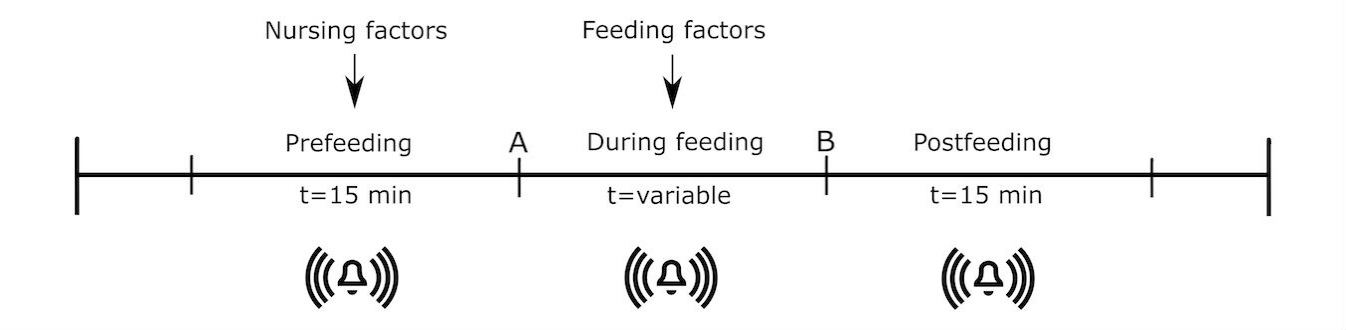
Each feeding episode is of variable duration with A and B representing the start and end time of feeding, respectively. Nursing care takes place in the period immediately before feeding. Alarms are analyzed for periods of prefeeding, during feeding, and postfeeding.

Cardiorespiratory events leading to patient monitoring alarms were automatically logged at the central monitors at the nursing station. These alarms were also displayed, in real time, on the patient monitors, the interbed communication system (which shows alarms originating from other beds at patient monitors), and the central monitors at the nursing station, in addition to being transmitted to handheld devices carried by the nurses. For this analysis, all critical patient monitor alarms corresponding to desaturation (SpO_2_ ≤80%) and bradycardia (heart rate ≤80 bpm) were measured. SpO_2_ was monitored using disposable pulse oximetry sensors (LNOP Neo PT-L or LNOP Neo, Masimo SET) with an average setting of 10 s and alarms being generated after a 10-s delay if the SpO_2_ fell to and remained below 80%. Bradycardia alarms occurred via heart rate measures using a 3-lead electrocardiogram (ECG) sensor (3M Red Dot or Ambu BlueSensor) with the heart rate being calculated as the average of the 12 most recent beat-to-beat intervals or the 4 most recent beat-to-beat intervals if the heart rate was less than 80 bpm. Alarms were generated as soon as the heart rate fell below 80 bpm. Other critical alarms were excluded, because they were found to constitute less than 15% of the total alarms [5]. In particular, apnea alarms based on chest impedance were excluded from the analysis, because recent research has indicated that apnea alarms are unreliable and are underdetected by monitors [12]. As shown in Figure 1, for every feeding episode, alarms in the 15 min before feeding (prefeeding period), the duration of feeding, and 15 min postfeeding were retained for analysis. These 15-min time windows were empirical choices based on unit experience.

To reaffirm the presence of the previously identified 2-hourly periodicity in alarms [5], alarm data from all infants present in the NICU during this study period were used to plot the average number of desaturation and bradycardia alarms through the course of the day. The number of alarms was normalized to represent the alarm pressure as would be generated by 100 infants for each consecutive 10-min interval of time [5].

### Exclusion Criteria

Exclusion criteria included both specific participants and specific instances of feeding. To account for interinfant and intrainfant variability, only those infants with 25 or more records of enteral feeding were retained for the final analysis. Feeding episodes from days on which infants were mechanically ventilated were excluded because of the invasive nature of mechanical ventilation that requires an endotracheal tube as opposed to the continous positive airway pressure mode of ventilation. Furthermore, those episodes of feeding that occurred when the infant was supine or received feeding via a combination of push and gravity were excluded, because these events occurred infrequently. Although enteral feeding during kangaroo care is promoted in our unit, the proportion of feeding episodes associated with kangaroo care in comparison with the total number of feeding episodes were small and were also excluded from analysis. Upon applying these criteria, 6.22% of 2282 feeding episodes were excluded.

### Statistical Modeling

The primary analysis, modeling the relationship between the dependent variable (alarms) and the independent variables (type of feeding, the position of an infant, etc.), was carried out using the generalized linear mixed model (GLMM), specifically logistic regression with mixed effects. With this model, 15 independent variables were investigated corresponding to the characteristics of the infants, such as feeding events and nursing care (Table 2). Details of this model are provided in Multimedia Appendix 1.

The *mixed* nature of the model arises from the presence of fixed and random effects. The fixed effects correspond to the regression terms that arise from independent variables and additionally due to any interaction between them (eg, type of feeding and position of infant). Random effects correspond to variable factors such as individual infants and each day within an infant and can, therefore, account for within-infant and within-day correlations. As an infant, in addition to exhibiting physiological patterns unique to oneself, may also have differences from one day to another; for example, a lasting physiological effect because of a recently performed medical procedure, random effects corresponding to the infant (RE-infant) and each day within an infant (RE-day) were incorporated. An interaction term was also included in the model to investigate the relationship between the position of the infant and the type of feeding. This decision was motivated by previous observations that position modulates physiology [13,14]

For the overall model, the statistical significance is reported. For each of the fixed effects and interaction terms, the regression coefficients were estimated along with the standard error, 95% CI, and statistical significance. For each random effect term, the standard deviation (SD) of its additive effect (by default, mean is zero) was estimated along with its 95% CI. For all statistically significant effects, the odds ratio (OR) and their 95% CI were reported. For the push and gravity methods of feeding, for both the prone and lateral positions of infants, the probability and SD of an alarm arising in response to feeding were estimated. These probabilities were calculated for the most representative values of our study population, that is, at median values.

In addition to the primary model that investigates the relationship between the variables listed in Table 2 and feeding-related alarms, subanalyses were carried out to specifically identify other relationships including the effect of left or right lateral position on feeding; whether feeding leads to desaturation alarms, bradycardia alarms, or both; and whether nursing care also leads to alarms. These subanalyses were carried out with the following combinations of dependent variables and fixed effects, while including RE-infant and RE-day: (1) presence or absence of desaturation and bradycardia alarms during and postfeeding for records corresponding to left and right lateral positions with all fixed effects, (2) individually testing for the presence or absence of desaturation and bradycardia alarms, one at a time, during and postfeeding with all fixed effects, and (3) presence or absence of desaturation and bradycardia alarms in the prefeeding period alone while including only nursing care events, postmenstrual age (PMA), and gender as fixed effects. All data were analyzed using Matlab R2015b Matlab (MathWorks). A *P* value of .05 was considered significant.

**Table 2:** The data types and distributions of all independent variables.

Independent variables	Data type	Distribution
PMA^a^	Categorical; ≤32 weeks or >32 weeks	1394 (65.14) ≤32 weeks
Gender	Categorical; male or female	976 (45.60) male
Type of feeding	Categorical; gravity or push	1251 (58.50) gravity
Nature of milk	Categorical; breast milk or formula	1636 (76.45) breast milk
Quantity of milk, mL per feed	Numerical^c^	15 (13-18)
Duration of enteral feeding, min	Numerical	10 (7-14)
Position of infant^b^	Categorical; lateral or prone	1078 (50.37) lateral
Diaper change	Categorical^d^; yes or no	618 (28.88)
Washing	Categorical; yes or no	98 (4.58)
Weighing	Categorical; yes or no	86 (4.02)
Airway suctioning	Categorical; yes or no	174 (8.13)
Change of nasal mask or nasal prong	Categorical; yes or no	487 (22.76)
Enteral administration of medication	Categorical; yes or no	222 (10.37)
Miscellaneous care	Categorical; yes or no	58 (2.71)
No nursing care	Categorical; yes or no	69 (3.22)

^a^PMA: postmenstrual age.

^b^All independent variables from *position of infant* until *no nursing care* were observed prefeeding.

^c^Numerical data are expressed as median (IQR).

^d^Categorical data are expressed as n (%) yes.

## Results

We analyzed critical alarms in 2140 nurse-annotated episodes of enteral feeding. A total of 19.77% of these episodes (423 episodes) had at least one critical alarm during and immediately after feeding. The data were acquired from 25 preterm infants, each of whom contributed a median of 77 feeding episodes (interquartile range [IQR], 55-114) during a median length of stay of 40 days (IQR, 22-70).

**Figure 2:**
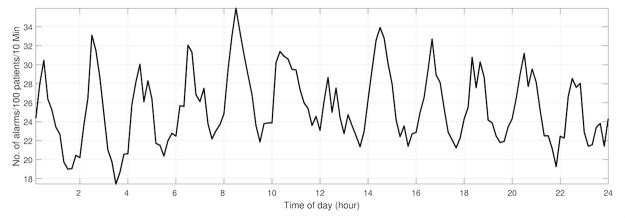
The average number of all desaturations and bradycardia alarms measured during the study period is plotted through the 24 hours of the day. The 2-hourly increase in alarms (at even hours) occurs at times scheduled for nursing care and enteral feeding. The resolution of the x-axis is 10 min. The y-axis represents the alarm rate for 100 patients per 10 min.

The 2-hourly increase in alarms, associated with the enteral administration of feeding and nursing care, is illustrated in Figure 2. The use of a GLMM allows for the analysis of feeding alone, whereas other factors (eg, nursing care) are held constant at arbitrary levels. The regression coefficients for the primary model including their 95% CI and statistical significance are detailed in Multimedia Appendix 2. First, the primary analysis shows that the position of infants significantly modulates the number of critical alarms generated by the push and gravity methods of feeding (*P*=.02, Multimedia Appendix 2). In addition to the presence of a statistically significant interaction between position and the types of feeding, the odds of alarms increase with every increasing minute of feeding (OR 1.02; 95% CI 1.004-1.04; *P*=.01).

**Figure 3:**
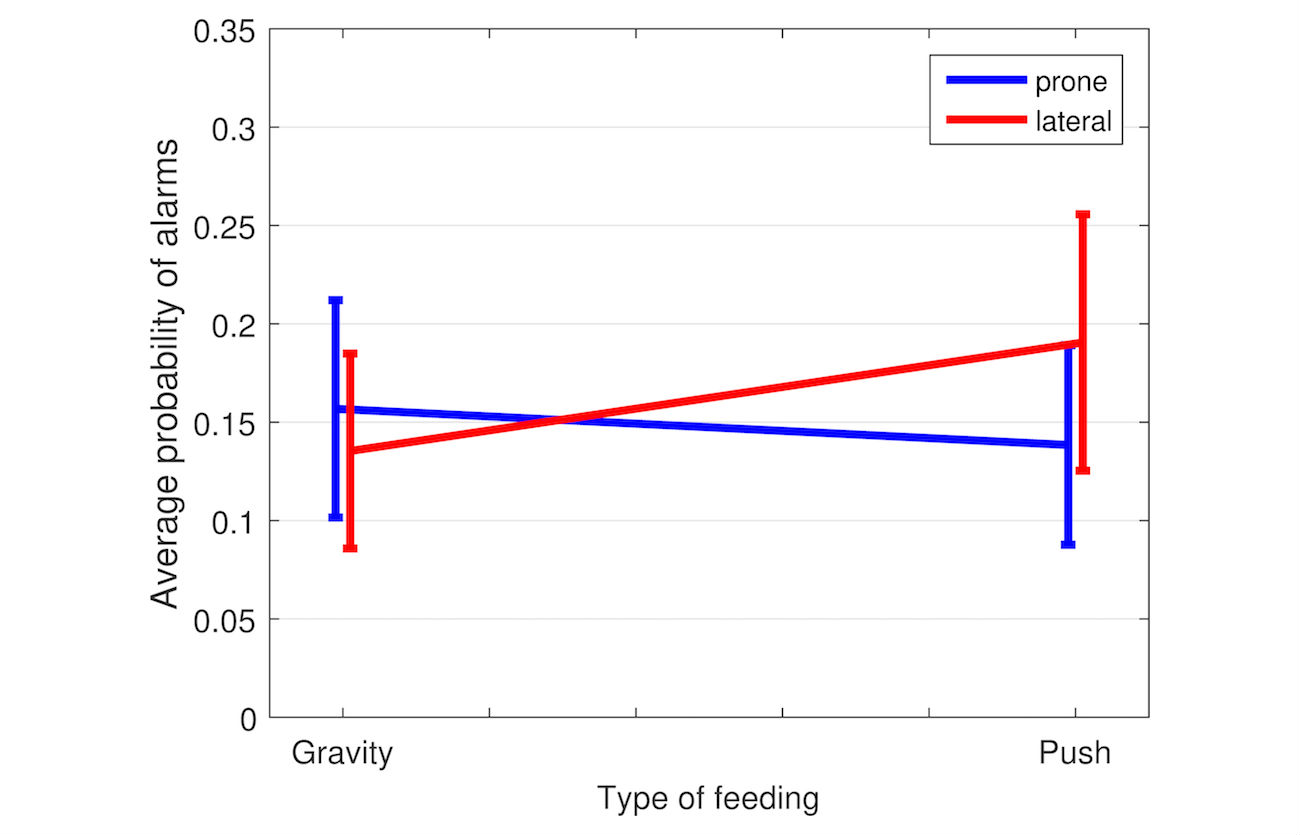
The probability of alarms in the prone (blue line) and lateral (red line) position for push and gravity types of feeding during and immediately after feeding. The intersection of the lines indicates an interaction between the position of the infant and the type of feeding. In the lateral position, the push method of feeding leads to a 40% increase in the probability of alarms in comparison with gravity (*P*=.02). The type of feeding does not affect the number of alarms when prone. The error bars indicate the standard deviation of the probability estimates.

Figure 3 illustrates the interaction between the position of the infant and the type of feeding. It shows that in the lateral position, at median levels of all other variables and without contributions from random effects, the push method of feeding leads to a 40% increase in the probability of alarms in comparison with gravity (*P*=.02). There is no change in the probability of alarms between push and gravity methods of feeding if the infant is prone (*P*=.42).

Second, a subanalysis of those feeding episodes during which the infant was in the left or right shows that push, irrespective of the left or right lateral position, increases the OR of an alarm by 1.7 (95% CI 1.1-2.6; *P*=.009) and that all other variables are not statistically significant.

Third, a subanalysis of only desaturation alarms from the feeding and postfeeding period shows that infants generate significantly more alarms in the lateral position when fed by the push method of feeding (*P*=.01), whereas, in the prone position, both methods of feeding produce the same number of alarms. In the case of bradycardia alarms, however, the position does not modulate the number of alarms generated by the different methods of feeding.

Finally, the primary model used for assessing desaturation and bradycardia alarms during and postfeeding is statistically significant with fixed effects alone (*P*<.001). The addition of random effects accounts for dependencies in the data, and their inclusion improves the fit of the model to the data (*P*<.001). The 25 coefficients (number of infants) corresponding to RE-infant and 308 coefficients (number of infant-days) corresponding to RE-day contributed to 8% and 7% of the total variance, respectively.

The subanalysis of the prefeeding period with nursing care events, PMA and gender as fixed effects, and desaturation and bradycardia alarms in the prefeeding period as the dependent variables (both random effects included) shows that changing nasal mask or nasal prong increases the OR of an alarm by 1.7 (95% CI 1.1-2.8; *P*=.02), whereas other variables are not statistically significant.

## Discussion

### Principal Findings

We found that the position of infants significantly modulates the incidence of cardiorespiratory alarms generated by the two methods of feeding after accounting for all other variables. When infants are in the lateral position, the push method of feeding generates 40% more alarms than gravity, whereas if the infant is prone, both methods of feeding generate a statistically similar number of alarms. With regard to the lateral position, being left or right lateral is inconsequential.

These results suggest that the physiological stability of infants can be improved by using the gravity method of feeding when infants are in the lateral position. The need for such noninvasive strategies for optimizing feeding in preterm infants has also been highlighted elsewhere [15,16]. Although decreasing critical cardiorespiratory alarms is a desirable goal in its right, research on extremely preterm infants has also shown an association between hypoxemic episodes and increased risk of late death or disability at 18 months of age [17]. Additionally, this simple measure can have implications for reducing alarm fatigue in nurses, a notable safety hazard that compromises clinical workflow and patient safety [18-21]. A reduction in alarms also implies a reduction in the loud transient noises that alarms generate, a known cardiorespiratory stressor for preterm infants. Such iatrogenic environmental hazards in the NICU have been proven to impair development in multiple ways, including possibly neurodevelopmental outcomes [7,9,22-25].

As seen from the subanalyses of desaturation and bradycardia alarms, position modulates the prevalence of desaturation alarms for the two types of feeding but not bradycardia alarms. This result suggests that desaturations that occur as a result of interaction between position and type of feeding are not because of critical episodes of bradycardia but maybe related to other pathophysiologies, such as apnea triggered by gastroesophageal reflux. The fact that push feeding increases desaturation alarms in the lateral position but not in the prone position may suggest the activation of a peripheral mechanism, such as laryngeal stimulation, perhaps because of reduced abdominal pressure or in response to gastroesophageal reflux while lateral, resulting in central apnea via chemosignaling.

In addition to the interaction between the position and type of feeding, the duration of feeding also leads to a statistically significant increase in alarms after accounting for all other variables. As the duration of feeding (and not the volume of milk) is a significant factor, every increased minute of feeding increases the odds of an alarm occurring, and this suggests that the stimulation of peripheral receptors during feeding is continuous and not just precipitated by the first moments of feeding.

The addition of random effects—RE-infant and RE-day—significantly improves the model, although the results show that interinfant and intrainfant contribution to the total variance is small. Furthermore, the inclusion of random effects strengthens the assumption of independence between individual feeding episodes and allows for the quantification of interpatient and intrapatient variability that could not be explained by the fixed effects of the model.

To observe the immediate effect of nursing care, applying the model to the prefeeding period (after excluding feeding-based variables) shows that changing the nasal mask or prong increases the odds of an alarm. This suggests that the procedure for changing nasal mask or prong is particularly stressful for the infant (other nursing care events have no effect in this period), as similar findings have been noted in other literature as well [26-28].

### Strengths and Limitations

A major limitation of the study is the observational nature of choice for push or gravity method of feeding based on the preference of nurses. This resulted in a quasi-random administration of feeds and was reflected in the balanced administration of push and gravity methods of feeding. Furthermore, the inclusion of random effects strengthens the assumption of independence between feeding episodes. Future studies attempting to reproduce this research will benefit from incorporating airflow sensors that can help determine whether desaturations in response to feeding occur because of apneas. The strengths of this study are the analysis of a large number of well-characterized feeding episodes of preterm infants in a NICU. The 2-hourly fluctuations in critical alarms support the earlier observation of feeding-associated instability [5]. The nature of the model employed here allows for the decomposition and quantification of every confounding factor that was incorporated into the model and to independently study their contribution, revealing that both nursing care (change of nasal mask or prong) and enteral feeding can lead to an increase in critical alarms.

### Conclusions

We recommend a strategy for reducing critical alarms associated with enteral feeding by choosing to feed those infants who are in the lateral position by the gravity method of feeding. For infants in the prone position, we found no evidence to recommend one method of feeding over the other.

## Appendix

Multimedia Appendix 1 Details of the logistic regression model with mixed effects.

Multimedia Appendix 2 A table of regression coefficients corresponding to the primary analysis using the mixed-effects logistic regression model.

## Abbreviations

bpm: beats per minute
ECG: electrocardiogram
GLMM: generalized linear mixed models
IQR: interquartile range
NICU: neonatal intensive care units
OR: odds ratio
PMA: postmenstrual age
RE: random effect
SpO2: oxygen saturation
SD: standard deviation
WMO: wet medisch-weten- schappelijk onderzoek
