# Supplementary material for: A Strategy to Reduce Critical Cardiorespiratory Alarms due to Intermittent Enteral Feeding of Preterm Neonates in Intensive Care
Source: Interact J Med Res. 2017 Oct 20;6(2):e20. doi: 10.2196/ijmr.7756 (PMC5670314; doi:10.2196/ijmr.7756)
Supplement: Multimedia Appendix 1 [file ijmr_v6i2e20_app1.pdf]

The logistic regression model with mixed effects

A specific case of the generalized linear mixed-models (GLMM), the logistic regression with fixed and random effects was employed for analysis. The dependent variable was binary and categorical, corresponding to the presence or absence of a critical bradycardia or desaturation alarm during the period corresponding to feeding and the 15 minutes post-feeding, and thus a Bernoulli distribution. The choice to binarize the number of critical alarms instead of using the absolute counts was motivated by the fact that typically there are no alarms, as would be expected for any short duration of time, leading to a skewed distribution that consists primarily of no alarms (zero-inflated) followed by a Poisson-like count distribution. Since such distributions are difficult to model using GLMM, the count data of alarms was binarized into presence/absence of alarms and modeled using logistic regression with 'logit' as the link function.

The regression coefficients were calculated using maximum likelihood estimates. The categorical variables were dummy coded using the 'reference' method and therefore in the case of interaction, the estimates for the main effects were not 'true' main effects but rather the effect of one variable at a specific level of the other variable. The significance of the interaction between position and type of feeding, e.g., the effect of gravity feeding when the infant is prone, etc. were tested using appropriate contrast matrices.

Furthermore, the inclusion of random effects strengthens the assumption of independence of individual feeding moments conditional on the random effects of infant and infant-days. The fit of the regression model to the data was tested using the F-test. The likelihood ratio test was used to check the goodness of fit of the model after incorporating a random effect, in comparison with a simpler model, e.g., a model with just fixed effects or with fixed effects and fewer random effect terms. Given the fact that the logistic function has a variance of $\pi^2/3$,

the contribution of the random effects terms to the overall variance ($\pi^2/3$ + sum of variance contributions of each random effect term) was calculated.
