# Supplementary material for: A Strategy to Reduce Critical Cardiorespiratory Alarms due to Intermittent Enteral Feeding of Preterm Neonates in Intensive Care
Source: Interact J Med Res. 2017 Oct 20;6(2):e20. doi: 10.2196/ijmr.7756 (PMC5670314; doi:10.2196/ijmr.7756)
Supplement: Multimedia Appendix 2 [file ijmr_v6i2e20_app2.pdf]

Regression coefficients corresponding to the primary analysis using the mixed-effects logistic regression model.

**Name of the regression coefficient**	**Estimate (SE)**	**95% CI**	***P*-value**
Intercept	-2.33 (0.48)	-3.27 to -1.38	<.001
PMA, ≤ 32 weeks	0.10 (0.20)	.61	
	-0.50 to 0.30		
Gender, male	0.08 (0.29)	-0.50 to 0.65	.79
Type of feeding, gravity	0.15 (0.18)	-0.21 to 0.50	.42
Nature of milk, expressed breast milk	0.003 (0.28)	-0.56 to 0.55	.99
Quantity of milk	0.02 (0.02)	-0.02 to 0.06	.27
Duration of feeding	0.02 (0.008)	0.004 to 0.03	.01
Position of infant, lateral	0.38 (0.19)	0.008 to 0.75	.04
Type of feeding : Position of infant	-0.55 (0.25)	-1.03 to -0.07	.02
Diaper change, yes	-0.22 (0.25)	-0.26 to 0.71	.36
Washing, yes	-0.17 (0.35)	-0.51 to 0.85	.62
Weighing, yes	0.09 (0.37)	-0.81 to 0.63	.81
Airway suctioning, yes	-0.07 (0.25)	-0.56 to 0.42	.77
Change of nasal-mask or nasal-prong, yes	0.25 (0.24)	-0.72 to 0.21	.29
Enteral administration of medication, yes	0.45 (0.23)	0.003 to 0.89	.05
No nursing care, yes	-0.13 (0.37)	-0.86 to 0.59	.72
Miscellaneous care, yes	0.03 (0.36)	-0.67 to 0.75	.91
RE-Infant	SD, 0.57	0.39 to 0.83	-
RE-Day	SD, 0.51	0.33 to 0.77	-

Abbreviations: SE, standard error; since the type of feeding interacts with the position of the infant, the SE, 95% CI and the P-value for these regression coefficients should not be interpreted standalone.
